# Silicon photonic Bessel–Gaussian beam generation unlocks new possibilities for long-range sensing

**DOI:** 10.1038/s41377-023-01189-0

**Published:** 2023-06-07

**Authors:** Sangsik Kim

**Affiliations:** School of Electrical Engineering, Korea Advanced Institute of Science and Technology, Daejeon, 34141 Republic of Korea

**Keywords:** Silicon photonics, Optical sensors

## Abstract

Concentrically distributed silicon photonic grating arrays generate long-range Besse–Gaussian beams, enabling rotational and range measurements over obstacles. This compact and mass-producible chip unlocks new potentials for long-range sensing and applications.

Bessel beams, characterized by their field distribution described by the first kind of Bessel function, exhibit unique oscillatory field behavior in the radial direction, providing a *non-diffractive* beam solution with an infinite depth of focus^1^. Notably, Bessel beams are also known as *self-healing* beams due to their ability to reconstruct their beam shapes even when partially interrupted by obstacles. These two distinct characteristics—*non-diffractive* and *self-healing*—make Bessel beams exceptionally suitable for long-range optical sensing applications, particularly in environments with obstacles and scattering.

Nevertheless, generating a true Bessel beam is practically limited by its radially unbounded oscillatory field, which necessitates infinite energy. Consequently, approximated Bessel beam solutions are predominantly employed in lieu of a radially unbounded ideal Bessel beam. A good example is the Bessel–Gaussian beam, where a Gaussian envelope function rapidly attenuates the Bessel beam’s slowly decaying oscillative radial components^2^. This hybrid beam effectively combines the properties of both Bessel and Gaussian beams and is more straightforward to generate than an ideal Bessel beam, for example, by illuminating an optical axicon with a Gaussian beam^3^.

Various methods have been explored for generating Bessel–Gaussian beams, including axicons^3^, holograms^4^, and spatial light modulators^5^. These traditional techniques rely on bulky optical elements, making them cumbersome and impractical for many field applications. To address these challenges, researchers have investigated alternative approaches such as metasurfaces^6^, photonic integrated circuits (PICs)^7^, and 3D-printed optical fibers^8^, primarily aiming to miniaturize the required components. However, these compact configurations have exhibited a limited depth of focus, typically spanning from hundreds of micrometers to tens of centimeters. Within these propagation distance ranges, even PIC-generated Gaussian beams with hundreds of micrometers in beam diameter have also been developed and employed^9,10^. Thus, for the practical use of a *non-diffractive* Bessel beam in long-range sensing, the development of a compact device, such as a PIC, capable of generating a Bessel–Gaussian beam with a depth of focus extending over tens of meters is crucial.

In this issue of *Light: Science & Applications*, Z. Zhi et al. at Jilin University report a groundbreaking silicon photonic chip capable of generating a Bessel–Gaussian beam with an impressive propagation distance of ≈10.24 meters, far surpassing previous approaches (Fig. 1)^11^. Their foundry-fabricated silicon chip incorporates concentrically distributed conventional grating couplers that emit Gaussian beams at the far-field, forming a Bessel–Gaussian beam through the superposition of 64 Gaussian beams from each grating. The device, with a footprint of ≈0.6 mm^2^, achieves a Bessel–Gaussian spot diameter (defined by the innermost ring’s diameter) ranging from 0.41 to 2.45 cm at distances between 1.55 and 10.24 meters from the chip. For a comparison with a typical Gaussian beam, the Rayleigh range for a beam diameter of 0.87 mm under the same conditions is only about 0.4 m. Distinctly, the researchers refrained from using additional optical lenses for these characterizations, and the reported operating wavelengths spanned from 1500 to 1630 nm, constrained by their tunable laser bandwidth.

**Fig. 1:**
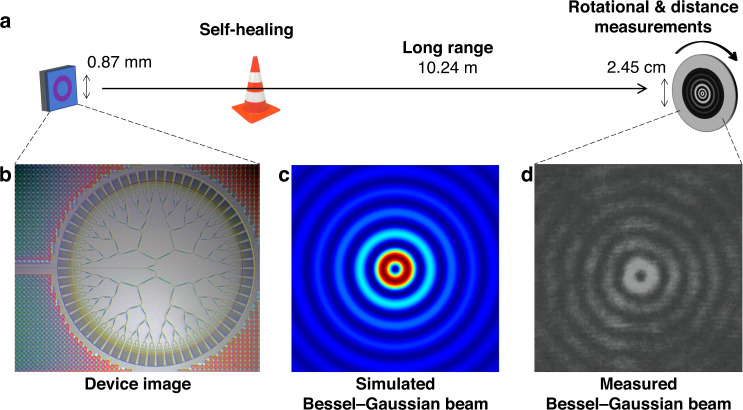
Illustration of the on-chip Bessel–Gaussian beam generation with long-range and self-healing characteristics for rotational and distance measurements. **a** Overall scheme, **b** Zoomed-in device image, and **c** Simulated and **d** Measured Bessel–Gaussain beams

Moreover, to demonstrate the functionality of the chip-generated Bessel–Gaussian beam, Z. Zhi et al. showed simultaneous rotation speed and distance measurements of a spinning object. They employed the rotational Doppler effect to measure the object’s rotation speed, characterizing different speeds of a bladeless optical chopper in the range of 75 to 100 r/s (i.e., 471 to 628 rad/s) while achieving a maximum error of 0.05%. To further highlight the *self-healing* characteristics of the Bessel–Gaussian beam, the team conducted identical rotation measurements, introducing a 2 mm-diameter copper wire as an obstacle between the source and the rotating object. Despite the reduced power in the signal, they successfully measured the object’s rotational speed with remarkable accuracy, highlighting the beam’s resilience.

This innovative method for generating long-range Bessel–Gaussian beams, utilizing a low-cost and mass-producible complementary metal–oxide–semiconductor (CMOS) process, offers immense potential for various long-range sensing applications beyond the laboratory setting. With a few technical enhancements, such as increasing power efficiency and introducing beam tunability, this Bessel–Gaussian beam generation approach could become even more versatile and suitable for practical applications. Furthermore, in conjunction with additional advances in photonic integrated circuits (PICs), including integrated light sources, detectors, and modulators, the on-chip generation of Bessel–Gaussian beams is expected to transform a wide range of applications in optical sensing, communication, manipulation, and beyond.
